# United States Acculturation and Cancer Patients’ End-of-Life Care

**DOI:** 10.1371/journal.pone.0058663

**Published:** 2013-03-11

**Authors:** Alexi A. Wright, Heather Stieglitz, Yankel M. Kupersztoch, M. Elizabeth Paulk, Yookyung Kim, Ingrid T. Katz, Francisco Munoz, Rachel B. Jimenez, Jan Mutchler, Lorna Rivera, Anthony L. Back, Holly G. Prigerson

**Affiliations:** 1 Department of Medical Oncology, Dana-Farber Cancer Institute, Boston, Massachusetts, United States of America; 2 Center for Psychosocial Epidemiology and Outcomes Research, Dana-Farber Cancer Institute, Boston, Massachusetts, United States of America; 3 Harvard Medical School, Boston, Massachusetts, United States of America; 4 Center for Outcomes and Policy Research, Dana-Farber Cancer Institute, Boston, Massachusetts, United States of America; 5 Parkland Center for Clinical Innovation, Dallas, Texas, United States of America; 6 University of Texas Southwestern Medical Center, Dallas, Texas, United States of America; 7 University of Pittsburgh School of Medicine, Pittsburgh, Pennsylvania, United States of America; 8 Department of Medicine, Brigham and Women’s Hospital, Boston, Massachusetts, United States of America; 9 Pomona Valley Hospital Medical Center, Pomona, California, United States of America; 10 Department of Radiation Oncology, Dana-Farber Cancer Institute, Boston, Massachusetts, United States of America; 11 University of Massachusetts Boston, Boston, Massachusetts, United States of America; 12 University of Washington, Fred Hutchinson Cancer Research Center, Seattle, Washington, United States of America; 13 Department of Psychiatry, Brigham and Women’s Hospital, Boston, Massachusetts, United States of America; Institut Gustave Roussy, France

## Abstract

**Background:**

Culture shapes how people understand illness and death, but few studies examine whether acculturation influences patients’ end-of-life treatment preferences and medical care.

**Methods and Findings:**

In this multi-site, prospective, longitudinal cohort study of terminally-ill cancer patients and their caregivers (n = 171 dyads), trained interviewers administered the United States Acculturation Scale (USAS). The USAS is a 19-item scale developed to assess the degree of “Americanization” in first generation or non-US born caregivers of terminally-ill cancer patients. We evaluated the internal consistency, concurrent, criterion, and content validity of the USAS. We also examined whether caregivers’ USAS scores predicted patients’ communication, treatment preferences, and end-of-life medical care in multivariable models that corrected for significant confounding influences (e.g. education, country of origin, English proficiency). The USAS measure was internally consistent (Cronbach α = 0.98); and significantly associated with US birthplace (*r* = 0.66, P<0.0001). USAS scores were predictive of patients’ preferences for prognostic information (AOR = 1.31, 95% CI:1.00–1.72), but not comfort asking physicians’ questions about care (AOR 1.23, 95% CI:0.87–1.73). They predicted patients’ preferences for feeding tubes (AOR = 0.68, 95% CI:0.49–0.99) and wish to avoid dying in an intensive care unit (AOR = 1.36, 95% CI:1.05–1.76). Scores indicating greater acculturation were also associated with increased odds of patient participation in clinical trials (AOR = 2.20, 95% CI:1.28–3.78), compared with lower USAS scores, and greater odds of patients receiving chemotherapy (AOR = 1.59, 95% CI:1.20–2.12).

**Conclusion:**

The USAS is a reliable and valid measure of “Americanization” associated with advanced cancer patients’ end-of-life preferences and care. USAS scores indicating greater caregiver acculturation were associated with increased odds of patient participation in cancer treatment (chemotherapy, clinical trials) compared with lower scores. Future studies should examine the effects of acculturation on end-of-life care to identify patient and provider factors that explain these effects and targets for future interventions to improve care (e.g., by designing more culturally-competent health education materials).

## Introduction

The provision of culturally sensitive medical care is likely to become an increasingly important issue as the Health Care Reform Act extends access to over 30 million previously uninsured Americans, many of whom will be immigrants. In 2010, 36% of the United States (US) population identified as racial or ethnic minorities, [1] and nearly 13% were foreign-born. [2] As the US becomes increasingly diverse, there is a growing need to understand how patients’ cultural beliefs and perspectives may inform their medical decision-making. Given that minorities tend to be diagnosed with cancer at more advanced stages compared with white patients,[3]–[7] there is a particular need to understand how acculturation affects medical decision-making in late-stage disease. This information is crucial for the provision of culturally-competent health education, informed medical decision-making, and the delivery of effective and equitable medical care.

In the US, racial and ethnic minorities receive fewer definitive cancer treatments and have lower five-year survival rates,[7]–[13] but receive more aggressive end-of-life care.[14]–[17] Healthcare disparities may be heightened in immigrants due to language barriers,[18]–[24] reduced access to care,[25]–[27] and/or different cultural values. [28], [29] Research has documented variations in treatment preferences and advance care planning among racial and ethnic minority groups.[30]–[32] To date, few studies have examined whether US acculturation influences cancer patients’ communication with physicians, treatment preferences, or medical care among first-generation and non-US born patients. To the extent that acculturation influences medical decision-making and care, it might help inform interventions to reduce ethnic disparities (e.g., by providing health information that is tailored to patients’ degree of US acculturation to ensure that patients are making well-informed decisions that are consistent with their cultural values and beliefs).

Acculturation describes a process by which the behaviors, values, beliefs, and identity of individuals from one culture are modified as a result of contact with another culture. [33], [34] Previous studies have found that higher US acculturation levels are associated with increased cancer screening, [4] receipt of recommended cancer therapies, [35] and advance care planning. [36] One major limitation of existing studies, however, is that most rely upon proxy measures of US acculturation (e.g., language, foreign birthplace) that cannot capture the range of behaviors, preferences, identity, values, and social interactions associated with acculturation. Although several unidimensional and bidimensional acculturation scales have been developed,[37]–[40] each has limitations, including an over-reliance upon language, validation within a single ethnic group, or the inclusion of sociodemographic characteristics as proxy measures. [34].

This report describes the development and validation of a new acculturation scale, the “United States Acculturation Scale: A Measure to Assess Americanization” (USAS). The primary aim of this study was to determine the reliability and validity of this scale, which was administered to caregivers of terminally-ill cancer patients, rather than patients, to minimize burden on dying patients. The secondary aim was to examine associations between caregivers’ US acculturation levels and advanced cancer patients’ communication with physicians, treatment preferences, advance care planning, and cancer treatment received (i.e. chemotherapy, clinical trials, or symptom management).

We hypothesized that patients with more “Americanized” caregivers would have stronger preferences for prognostic information because they would feel better able to understand and use this information and because prognostic disclosure is more common in the US than other countries.[41]–[44] We also hypothesized that patients with more acculturated caregivers would be less likely to want intensive life-prolonging care at the end-of-life, [43] compared with patients whose caregivers were less acculturated. We expected more “Americanized” caregivers might have past experiences with intensive medical care that might enable them to evaluate better the risks and benefits of this intensive care at the end-of-life. [45] Less Americanized caregivers were expected to have values consistent with more religious and less educated ethnic minority patients whom we have shown to prefer more intensive end-of-life care. [46], [47].

## Methods and Findings

### Ethics

The institutional review boards of the Dana-Farber/Partners Cancer Center, Yale University, Parkland Hospital, West Haven Veterans’ Affairs Connecticut Comprehensive Cancer Center Clinic, Parkland Hospital, Simmons Comprehensive Cancer Center, and New Hampshire Oncology-Hematology approved all study procedures.

### Study Sample

Our data were collected as part of the Coping with Cancer study, a National Cancer Institute and National Institute of Mental Health-funded prospective, multi-institution cohort study of terminally ill cancer patients and their unpaid, informal caregivers (e.g., spouse, adult child). Participants were recruited from September 2002 to February 2008 from three academic institutions, a Veterans’ Administration hospital, “safety-net” center that serves many non-native citizens, and a private practice: Yale Cancer Center (New Haven, Connecticut), Simmons Comprehensive Cancer Center (Dallas, Texas), Dana-Farber/Partners Cancer Center (Boston, Massachusetts), the West Haven Veterans’ Affairs Connecticut Comprehensive Cancer Center Clinic (West Haven, Connecticut), Parkland Hospital (Dallas, Texas), and New Hampshire Oncology-Hematology (Hooksett and Londonderry, New Hampshire).

Eligibility criteria included: diagnosis of advanced cancer (distant metastases and disease refractory to first-line chemotherapy); age ≥20 years; presence of an informal caregiver; and clinic staff/interviewer assessment that the patient had adequate stamina to complete the interview. Patient-caregiver dyads in which either the patient or caregiver refused to participate, met criteria for dementia or delirium (by neurocognitive status examination), or did not speak either English or Spanish were excluded. All study participants provided written informed consent and were given a choice to be interviewed in English or Spanish. Interviews were conducted in English or Spanish by bilingual interviewers who were trained by the bilingual and bi-cultural scale developers. Each interviewer was required to meet study standards (e.g., >80% agreement between the scale developers and the trainee on the rating of how acculturated/“Americanized” the caregiver was) before being permitted to collect data for the study. Patients were followed from enrollment to death, and the caregivers were followed to a final study assessment of approximately 6 months after the death.

Of the 917 eligible participants, 638 patient-caregiver dyads (69.6%) consented and enrolled in the larger study. Of the 279 patients who refused participation, 120 were not interested, 69 cited other reasons, and 37 had caregivers who refused. There were no differences in the sociodemographic characteristics between participants and non-participants, except participants were more likely to be Hispanic (11.8% vs. 5.7%, p = 0.006). For the present analysis we restricted our sample to the 171 patient-caregiver dyads in which both identified as either non-US-born or first generation American, and the caregiver completed the acculturation scale. This group did not differ significantly (p<0.05) from the larger sample by gender, cancer type, performance status, or caregiver relationship (e.g. spouse, adult child). However, as expected, non-US-born and first generation Americans were more likely to have characteristics associated with recent immigration (e.g., be younger, non-native English speakers, born outside of the US, less educated, uninsured, and identify as Hispanic). The USAS was administered to caregivers of terminally-ill cancer patients as a proxy for patients’ degree of acculturation in order to minimize participant burden in this sample of dying patients.

### Measures

#### The United States Acculturation Scale

The USAS scale (Table S1) was developed from a literature review and the authors’ experiences caring for cancer patients at Parkland Hospital, a “safety-net” center that serves many non-native citizens, over the past decade. It was also informed by two of the scale’s developers’ (HS, YMK) experiences as dual citizens of the US and Mexico. The USAS scale assesses the degree of acculturation to American life with 19 items that measure language preferences and proficiency and cultural identity (e.g., music, entertainment, and food preferences; contact with English-language mass media; friendships, social interactions with US-born residents compared with others, frequency of contact with country of origin, and burial site preferences). The scale differentiates between five levels of acculturation–(1) Non-American only, (2) mostly non-American, (3) bicultural, (4) mostly American, and (5) American only–measured on a five-point Likert scale with higher scores reflecting greater “Americanization.” Scores on each scale item were totaled and divided by the number of items. In addition, the scale included the trained bilingual interviewer’s assessment of the respondent’s degree of acculturation, rated on a five-point Likert scale from “Completely non-American” to “Completely Americanized.”

US acculturation was measured in caregivers instead of patients to limit subject burden because our population of interest consisted of terminally-ill cancer patients. Although acculturation levels may differ between patients and caregivers, caregiver participants were identified by patients as “the person who provides you with the most *unpaid* assistance with your activities of daily living; e.g. bathing, cooking, transportation, housework, etc.” As such, we expected that enrolled caregivers would be the people whom patients depended upon most, and therefore would likely be influential participants in patients’ medical decision-making. Patient acculturation was assessed with the five item Cuellar’s validated “Acculturation Rating Scale for Mexican Americans” (ARS) short-form, [33] to minimize respondent burden in terminally ill patients. The five items, selected from the 20-item ARS, measured participants’ language preferences (e.g., in speech, writing, and thought) and language(s) spoken at home and with friends during childhood and now. Items were coded on a five-point Likert scale which ranged from “only non-English” to “only English,” with higher scores reflecting a stronger English language/Anglo orientation.

#### Sociodemographic characteristics

Participants’ age, gender, race/ethnicity, marital status, education, health insurance, and religious affiliation were recorded as reported. Race or ethnicity was determined by patient self-report in response to the open-ended question: “What race or ethnicity do you consider yourself to be?” Patients who identified as Hispanic were analyzed as such, regardless of race. Patients were specifically asked for their birthplace and native language. Caregivers provided similar sociodemographic information and their kinship relationship to the patient (e.g. spouse, adult child).

#### Disease characteristics and health status

Research staff reviewed the medical record and verified each patient’s primary cancer, stage, and performance status with the treating physician. Patients’ performance status and co-morbid medical conditions were assessed with the Karnofsky score and Charlson Comorbidity Index, respectively. [48], [49] Patients’ symptom burden was measured with the McGill Quality of Life Index’s physical health subscale. [50].

#### Patient-Physician communication

Because previous studies have documented differences in physicians’ affective and instrumental communication with ethnic minorities compared with white patients, [51] we sought to determine whether communication processes differed by acculturation level. Patients were asked in yes/no questions if they trusted their physician, whether doctors treated them with respect, and whether they had discussed their wishes for the type of care they would want to receive if dying with their physician. Patients were also asked: “How comfortable are you asking your doctor questions about your care?” using a five-point Likert scale (very uncomfortable to very comfortable).

#### Treatment preferences and planning

In the baseline interview patients were asked, “If your physician knew how long you had left to live, would you want him or her to tell you?” and “Would you want to be kept alive if it required being on a feeding tube?” Patients were also asked: “Do you think it would be a bad thing for a person to die in the ICU versus elsewhere (e.g., home, hospital, or hospice).”Response options were “yes” or “no.” Finally, patients were asked, “If you could choose, would you prefer: 1) a course of treatment that focused on extending life as much as possible, even if it meant more pain and discomfort, or 2) a plan of care that focused on relieving pain and discomfort as much as possible, even if that meant not living as long?” Response options were “extend life as much as possible,” “relieve pain or discomfort as much as possible,” or “don’t know.”

#### Treatments

Active treatments (palliative chemotherapy, radiation, pain management, and participation in clinical trials) were abstracted from the medical record and verified with the treating oncologist.

### Statistical Analyses

Cronbach’s α was used to evaluate the scale’s internal consistency (reliability) and to identify items that, if removed, would improve it. Principal component analyses produced item-factor loadings and eigenvalues for emergent factors. *t* tests and analysis of variance were used, depending upon whether the outcome was continuous or categorical, to examine associations between patients’ sociodemographic and health characteristics and caregivers’ acculturation level (e.g., caregivers’ mean USAS for patients who had health insurance, compared with those who did not).

Pearson correlation coefficients were used to estimate relationships between the scale and patients’ birthplace (United States vs. other), native language (English vs. other); and language proficiency (choice of English vs. Spanish for interview) as a measure of the scale’s concurrent validity. Criterion/construct validity was assessed by examining correlations between the caregivers’ USAS scale scores and: 1) patients’ scores on Cuellar’s validated ARS and 2) the rater’s perception of caregivers’ acculturation. A linear regression model estimated the unique contributions of the language and cultural identity items on the rater’s perception of caregivers’ acculturation level.

To assess predictive validity, multivariable regression models estimated the effects of caregivers’ acculturation on patient-physician communication, treatment preferences, and cancer treatment at baseline, a median of 5.8 months before death. Every variable that was associated (*p*<0.20) with both the predictor (caregiver acculturation) and outcome (e.g. treatment) was investigated as a potential confound and retained if significant (*p*≤0.05). Statistical analyses were performed with SAS software (version 9.2; SAS Institute, Inc, Cary, NC).

## Results

Table 1 shows that the USAS had a high degree of internal consistency (Cronbach’s α = 0.98). All questions had high item-total correlations, except for contact with country of origin (*r* = 0.27), which was retained because it captured a unique behavioral measure of acculturation and did not compromise the scale’s overall internal consistency. A principal components analysis of the 19-item USAS revealed a single emergent factor (eigenvalue = 14.22) that accounted for 79.0% of the variance in the data; a review of the scree plot further confirmed the presence of a single factor (data not shown).

**Table 1:** United States acculturation scale.

	Correlation Analyses		
Scale Items	Item with Total	Item with Rater Assessment^a^	Cronbach’s α 0.98
**Language**			
What language do you speak most often?^1^	0.95	0.90	
What language do you prefer speaking?^1^	0.95	0.90	
What language do most of your friends speak?^1^	0.93	0.87	
You are most comfortable reading (newspapers, books, magazines) in^1^	0.94	0.88	
You are most comfortable writing in^1^	0.94	0.89	
You think most often in^1^	0.94	0.88	
**Cultural Identity**			
The music you listen to most is in^1^	0.90	0.84	
The music you enjoy most is in^1^	0.91	0.82	
The TV shows you watch most are in^1^	0.90	0.83	
The TV shows you prefer watching most are in^1^	0.92	0.86	
The movies you watch most are in^1^	0.88	0.82	
Your father’s cultural identity was/is (country of origin)^2^	0.58	0.58	
Your mother’s cultural identity was or is (country of origin)^2^	0.72	0.70	
Your friends while you were growing up were of ______origin^2^	0.81	0.75	
Your family cooks/eats foods that are of _______ origin^2^	0.82	0.74	
Your friends now are of _______ origin^2^	0.88	0.79	
You like to identify yourself as^2^	0.87	0.83	
Where would you want to be buried?^3^	0.61	0.67	
Your contact (letters, phone calls, emails) with country of origin has been^4^	0.27	0.23	

a Dependent variable was the interviewer’s evaluation of how “Americanized” the respondent was. Response options included Likert scales (scored 1–5):

1 Non-English only to English only;

2 Non-American to American;

3 (Non-United States) country of origin vs. United States; and ^4^>twice/yr, >4 times/yr, monthly, weekly, or daily.

As shown in Table 2, patients whose caregivers had scores indicating lower acculturation levels were more likely to identify as Hispanic and Catholic, have fewer years of formal education, be uninsured, have been born outside of the US, and have lower Karnofsky scores, compared with patients with more acculturated caregivers. There were also significant differences in acculturation levels by institution; e.g., caregivers’ USAS scores were significantly lower at Parkland Hospital and Simmons Comprehensive Cancer Center, compared with the Yale Cancer Center. Caregivers were highly involved in patients’ care; 62% reported providing more than 80% of patients’ informal care. There were no differences in the type of relationships between patients and caregivers, or the extent of informal care provided by acculturation status.

**Table 2:** Patient characteristics by caregiver acculturation level (N = 171).

	Full Sample	Caregiver USAS	
Patient Characteristics^a^	N (%)	Mean (SD)	P-value
**Male**	85 (52.2)	3.5 (1.5)	0.62
**Age, years**			0.12
<50	47 (27.5)	3.2 (1.6)	
50–64	62 (36.3)	3.6 (1.4)	
>65	54 (31.6)	3.8 (1.2)	
**Race/ethnicity**	**<0.0001**		
White	79 (48.5)	4.5 (0.7)	
Black	8 (4.9)	4.7 (0.4)	
Hispanic	70 (42.9)	2.3 (1.1)	
Other	6 (3.7)	3.8 (0.7)	
**Married**	116 (72.5)	3.6 (1.4)	0.99
**Education, years**	**<0.0001**		
<8	53 (32.5)	2.4 (1.2)	
8–12	39 (23.9)	4.0 (1.3)	
>12	71 (43.6)	4.1 (1.0)	
**Health insurance**			**<0.0001**
Insured	103 (63.2)	4.3 (0.9)	
Uninsured	60 (35.1)	2.3 (1.1)	
**Native language** b			**<0.0001**
English	95 (55.6)	4.4 (0.8)	
Non-English	68 (40.0)	2.4 (1.2)	
**Nativity**			**<0.0001**
US-born	96 (56.1)	4.3 (0.9)	
Foreign-born	67 (39.2)	2.4 (1.3)	
Mexico	42 (24.6)	1.9 (0.9)	
Central/South America	10 (5.9)	2.1 (1.0)	
Europe	9 (5.3)	4.1 (1.0)	
South/SouthEast Asia	4 (2.3)	3.5 (0.6)	
Other	2 (1.2)	4.4 (0.7)	
**Cancer type**	0.08		
Breast	15 (8.8)	3.5 (1.3)	
Gastrointestinal	60 (35.1)	3.5 (1.4)	
Lung	27 (15.8)	4.1 (1.2)	
Other^c^	59 (34.5)	3.3 (1.5)	
	**Full Sample**	**Caregiver USAS** a	
**Patient Characteristics**	**N (%)**	**Mean (SD)**	**P-value**
**Karnofsky** d	**0.0004**		
>70	94 (55.0)	3.9 (1.3)	
<70	66 (38.6)	3.1 (1.5)	
**Religion**	**0.0005**		
Catholic	90 (52.6)	3.2 (1.4)	
Protestant	24 (14.0)	4.5 (0.8)	
Other	41 (23.9)	3.6 (1.3)	
None	8 (4.7)	4.2 (0.9)	
**Institution**	**<0.0001**		
Yale	62 (36.3)	4.4 (1.0)	
VA	5 (2.9)	4.9 (0.1)	
Simmons	6 (3.5)	3.1 (0.6)	
Parkland	65 (38.0)	2.3 (1.1)	
DFCI/MGH	15 (8.8)	4.0 (0.8)	
NHOH	17 (10.0)	4.2 (0.7)	
**Caregiver relationship**			0.15
Spouse	85 (49.7)	3.7 (1.3)	
Adult child	31 (18.1)	3.5 (1.4)	
Other family member or friend	33 (22.1)	3.1 (1.4)	
**Caregiver identifies as primary** e	154 (96.3)	3.6	0.25
**Unpaid, informal care provided by caregiver**			0.89
** ** ≥80%	101 (62.7)	3.6 (1.4)	
41–79%	51 (31.1)	3.6 (1.4)	
≤40	9 (5.5)	3.2 (1.4)	

a Missing data: male, age, race/ethnicity, education, health insurance, native English speaker, nativity, and religion (n = 8, 4.7%); marriage (n = 11, 6.4%), cancer type (n = 10, 5.8%), Karnofsky score (n = 11, 6.4%), caregiver relationship (n = 22, 12.9%), caregiver identifies as primary caregiver for patient (n = 4).

b Participants were asked whether English was their native language with response options of “yes” or “no.” Study participation mandated English or Spanish fluency, and interviews were conducted in English or Spanish by trained, bilingual research assistants.

c Other cancers each representing <5% sample.

d Karnofsky score is a measure of functional status that is predictive of survival, where 0 is dead and 100 is perfect health. The sample median, 70, reflects an ability to care for self, but not carry on normal activity or work.

e Caregivers were asked: "Do you consider yourself to be the patient's primary caregiver, defined as a family member or friend who provides the patient with *unpaid* assistance with his/her activities of daily living (e.g., bathing, cooking, transportation, housework etc). Caregivers who answered "yes" are included in this category.

Caregivers’ USAS language and cultural identity scores were closely and highly statistically significantly associated with patients’ birthplace, native language, and English proficiency, as shown in Table 3. They were also closely associated with patients’ scores on the Cuellar Brief Acculturation Scale (*r* = 0.65 and *r* = 0.60, p<0.0001, respectively), although 46.6% of cancer patients did not complete this measure, which validated our concern about administering the USAS to terminally ill patients. Caregivers’ English proficiency was also closely associated with their USAS scores (*r* = 0.86, p<0.0001 for full scale), and the raters’ assessment of caregivers’ degree of acculturation (*r* = 0.86, p<0.0001 for the full scale).

**Table 3:** Summary of validity analyses for caregiver United States acculturation scale.

	Language	Cultural Identity	Full Scale
Correlation with:	*Pearson r*	*Pearson r*	*Pearson r*
United States birthplace (patient)	0.67	0.67	0.66
Native English speaker (patient)	0.73	0.72	0.71
Interview conducted in English (patient)	0.74	0.73	0.71
Brief Acculturation Scale (patient)	0.65	0.60	0.64
Interview conducted in English (caregiver)	0.78	0.77	0.75
Rater assessment of acculturation (caregiver)	0.88	0.87	0.86

All P-value<0.0001; Missing data: United States birthplace and native English speaker (patient: n = 8, 4.7%), interview conducted in English (patient and caregiver: n = 9, 5.3%), Brief Acculturation Scale (patient: n = 80, 46.6%), and rater assessment of acculturation (caregiver: n = 4, 2.3%).

Table 4 shows that items measuring caregivers’ language and cultural identities were independently and highly statistically significantly associated with raters’ assessments of caregivers’ acculturation level. These results suggest that USAS’ items measuring caregivers’ cultural identity were distinct from those that measured caregivers’ language preferences and proficiency, and that each uniquely contributed to independent raters’ assessment of caregivers’ level of US acculturation.

**Table 4:** Associations between rater assessment of caregiver acculturation and language and cultural identity items.

	Rater Assessment of Caregiver Acculturation	
	Standardized β Coefficient^a^	P-value
**Language**	0.57	<0.0001
**Cultural Identity**	0.37	0.0009

a Data are expressed as standardized coefficients that reflect the unique contribution of each predictor (language or cultural identity) on the outcome (rater’s assessment of caregivers’ acculturation).

As shown in Table 4, patients whose caregivers had scores indicating lower acculturation levels were more likely to identify as Hispanic and Catholic, have fewer years of formal education, be uninsured, have been born outside of the US, and have lower Karnofsky scores, compared with patients with more acculturated caregivers. There were also significant differences in acculturation levels by institution; e.g., caregivers’ USAS scores were significantly lower at Parkland Hospital and Simmons Comprehensive Cancer Center, compared with the Yale Cancer Center.

As shown in Table 5, every unit increase in the caregiver’s USAS score was associated with a 1.3 fold increase in the cancer patient’s likelihood of wanting prognostic information (AOR = 1.31, 95% CI 1.00–1.72) and higher odds of wanting to avoid dying in an ICU (OR = 1.36, 95% CI 1.05–1.76). Higher USAS scores were also associated with decreased odds of wanting a feeding tube to extend life (AOR = 0.68, 95% CI 0.49–0.99). We did not detect any associations between caregivers’ acculturation and measures of patients’ trust in physicians, comfort asking questions about their care, or communication processes after adjustment for other confounds.

**Table 5:** Associations between Caregiver Acculturation and Patient-Physician Communication and Patients’ Treatment Preferences (N = 171).

Outcome Measure:	Full Sample		Caregiver USAS Score				
Patient Communication and Preferences	N	%	OR	(95% CI)	AOR		(95% CI)
**Patient-Physician Communication**							
Treated with respect^a^	162	94.7	–a	–a	–a		–a
Comfort asking questions about care^b^	123	71.9	**1.57**	**(1.21–2.03)** ***	1.23		(0.87–1.73)
End-of-life discussion with physician^c^	38	22.2	0.82	(0.63–1.06)	0.97		(0.71–1.31)
**Treatment Preferences**							
Prognostic information^d^	113	66.1	**1.36**	**(1.06–1.74)** **	**1.31**	**(1.00–1.72)** *	
Life-extending care over comfort^e^	47	27.5	1.13	(0.86–1.48)	1.16	(0.85–1.57)	
Avoid death in intensive care unit^f^	53	31.0	**1.36**	**(1.05–1.76)** *	**1.36**	**(1.05–1.76)** *	
Feeding tube to extend life^g^	55	32.2	0.80	(0.64–1.02)†	**0.68**	**(0.49–0.99)** *	

† p≤0.10.

* p≤0.05.

** p≤0.01.

*** p≤0.001.

Logistic regression models examined associations between acculturation and patient-physician relationships, treatment preferences, terminal illness acceptance, and advance care planning. Every variable that was associated (p<0.20) with both the predictor and outcome (e.g., age, ethnicity, education, health insurance, native English language, nativity, cancer type, performance status, religion, institution, survival, and caregiver relationship) was investigated as a potential confounder and retained if significant at a level of p<0.05. Models adjusted for:

a estimate for measures examining respect and trust in physicians (latter not shown) could not be calculated due to near uniform response (yes),

b Foreign born,

c performance status,

d spousal caregiver,

e performance status.

f no variables met significance (p≤0.05), and ^g^ age.

Missing data: treated with respect, comfort asking questions (n = 8, 4.7%); end of life discussion (n = 9, 5.3%); prognostic information (n = 12, 7.0%); life-extending care over comfort (n = 29, 17.0%); avoid death in intensive care unit (n = 14, 8.2%); and feeding tube to extend life (n = 13, 7.6%).

Associations between advanced cancer patients medical care and caregivers’ acculturation are shown in Table 6. For every unit increase in the caregiver’s USAS score, the patient’s odds of participating in clinical trials doubled (OR = 2.20, 95% CI 1.28–3.78). We investigated possible confounding influences, including native English language, educational level, patient performance status, and institution on the association between acculturation and trial participation, but did not find any evidence of confounding. Similarly, patients’ odds of receiving palliative chemotherapy increased approximately 60% for every unit increase in the caregiver’s acculturation level (AOR = 1.60, 95% CI 1.20–2.12).

**Table 6:** Associations between Caregiver Acculturation and Patient Medical Care (N = 171).

Outcome Measure:	Full Sample		Caregiver USAS Score			
Patients’ Medical Care	N	%	OR	(95% CI)	AOR	(95% CI)
Clinical trial^a^	22	12.9	**2.20**	**(1.28–3.78)** **	**2.20**	**(1.28–3.78)** **
Palliative chemotherapy^b^	97	56.7	**1.39**	**(1.11–1.75)** **	**1.59**	**(1.20–2.12)** **
Pain management exclusively^c^	53	31.0	**0.62**	**(0.49–0.80)** ***	0.75	(0.53–1.07)

** p≤0.01.

*** p≤0.001.

Logistic regression models examined associations between acculturation and patient-physician relationships, treatment preferences, terminal illness acceptance, and advance care planning. Every variable that was associated (p<0.20) with both the predictor and outcome (e.g., age, ethnicity, education, health insurance, native English language, nativity, cancer type, performance status, religion, institution, survival, and caregiver relationship) was investigated as a potential confound and retained if significant at a level of p<0.05. Models adjusted for:

a no variables met significance (p≤0.05) criteria for adjustment,

b age and cancer type, and^ c^ spousal caregiver.

Missing data: clinical trial (n = 17, 9.9%), palliative chemotherapy (n = 14, 8.2%), and pain management (n = 16, 9.4%).

## Discussion

The goal of this study was to develop and validate a measure of “Americanization” that could be used to assess acculturation in non-US born or first generation American respondents, independent of their country of origin. Our results demonstrate that the United States Acculturation Scale (USAS) is a reliable and valid measure of “Americanization” in a sample of terminally-ill cancer patients and their informal caregivers from Mexico, Central and South America, Europe, and Asia.

In this study, the USAS was closely associated with US-birthplace, English proficiency, and a trained rater’s evaluation of the respondent’s degree of Americanization, each of which provides evidence of the USAS’ criterion validity. Our analyses also demonstrate the predictive validity of the USAS which was associated with terminally ill cancer patients’ desire for prognostic information, end-of-life care preferences, and medical care. In particular, scores indicating greater acculturation are associated with increased odds of patients participating in clinical trials and receiving palliative chemotherapy, compared with scores indicating lower acculturation levels.

We found that advanced cancer patients’ preferences for information and specific end-of-life treatments varied by caregivers’ acculturation level. Specifically, patients with more acculturated caregivers were more likely to want prognostic information, compared to those with less acculturated caregivers, but were not more likely to have discussed their end-of-life wishes with a physician. Previous studies have documented an association between US acculturation and positive attitudes towards prognostic disclosure within Mexican-American and Japanese cultures. [43], [52], [53] Interestingly, we also did not observe any differences in patients’ comfort asking physicians’ questions about their medical care by caregivers’ acculturation level. Patients with more acculturated caregivers were also less likely to want a feeding tube to extend life or to die in an intensive care unit, compared with those with less acculturated caregivers. This is not surprising given that cultural attitudes likely influence how people understand their illness and therefore the medical choices made at the end-of-life. [54] Our finding that patients with less acculturated caregivers were more likely to prefer feeding tubes at the end-of-life may be a reflection of cultural norms, beliefs and values placed on the provision of food and nutrition by members of many ethnic/racial minority groups. [36], [55].

Other researchers have argued that English language proficiency predicts health care usage more than acculturation. [27] In this study, language did not explain the effect of acculturation on terminally ill cancer patients’ treatment preferences or end-of-life medical care. Language alone does not appear to capture the broader, more complex cognitive, emotional, and behavioral components of acculturation. This finding has important implications for intervention development because it suggests that direct translations of clinical materials (e.g., chemotherapy consent forms or educational materials) may be less effective than carefully crafted documents that address these issues within a larger cultural context that accounts for patients’ and caregivers’ degree of US acculturation. It also suggests that that increasing access to patient navigators who can act as cultural brokers may be a more promising approach for creating more culturally sensitive cancer care than would the use of medical interpreters. We believe an important conclusion of this study is that acculturation as a construct involves more than simply a recognition of linguistic differences, and cultural competence requires more than bilinguistic proficiency.

Racial and ethnic disparities in clinical trial participation have been reported previously, [56], [57] [7], [58], [59] but this is the first study, to our knowledge, to demonstrate that acculturation levels may influence advanced cancer patients’ enrollment in investigational trials and receipt of palliative chemotherapy. Several factors might explain this association. More acculturated patients may have greater access to institutions offering clinical trials, greater inherent trust in Western medicine, a higher likelihood of being referred for trials within institutions due to physicians’ biases, [60], [61] more experience navigating complex health care systems, and/or fewer logistical barriers (e.g., language difficulties complicating recruitment, travel, childcare responsibilities, inflexible work-hours). Similarly, less acculturated may have more difficulty understanding the rationale for non-curative chemotherapy or obtaining treatment. Future studies should examine the effects of acculturation on trial participation and use of non-curative chemotherapy in larger samples within institutions where clinical trials and patient navigators are readily available to determine the patient and provider factors that may mediate or moderate the effect of acculturation on cancer care at the end-of-life.

This study has several limitations. Although the study recruited participants from six different institutions, the sample size was small, less acculturated participants were predominantly recruited from Parkland Hospital, and the study eligibility criteria required that participants speak Spanish or English, which may limit the generalizability of our results outside of these populations. In addition, respondents were asked to self-report a single race/ethnic status, leaving uncertain the race of respondents who reported being Hispanic. We also used caregiver proxies to measure patients’ US acculturation status in order to limit subject burden because our population of interest consisted of terminally-ill cancer patients; although indirect, enrolled caregivers were highly involved in patients’ care (both by patient and caregiver report), and therefore were likely to be involved in medical decision-making. In addition, caregivers’ acculturation scores were highly correlated with patients’ scores on the Cuellar Brief Acculturation Scale, suggesting that they were reasonable proxies. Finally, the cross-sectional design of our study does not allow us to evaluate causality. Future studies should directly examine how the degree of “Americanization” influences patients’ understanding of their illness, communication with physicians, medical choices, and end-of-life care in larger, more ethnically diverse samples over time.

Our study describes a new multi-dimensional measure of acculturation which predicts advanced cancer patients’ end-of-life preferences, planning, and care. The provision of culturally sensitive care is likely to become an increasingly important issue as the Health Care Reform Act extends access to over 30 million previously uninsured Americans, many of whom will be immigrants. Given the associations between acculturation and end-of-life medical decision-making, our results suggest that health care providers should be attentive not only to the cultural differences embedded in racial and ethnic groups, but also to what extent patients and their families have become “Americanized.”

## Supporting Information

Table S1 **United States acculturation scale.**
(DOCX)
