# Supplementary material for: United States Acculturation and Cancer Patients’ End-of-Life Care
Source: PLoS One. 2013 Mar 11;8(3):e58663. doi: 10.1371/journal.pone.0058663 (PMC3594172; doi:10.1371/journal.pone.0058663)
Supplement: Table S1 — United States acculturation scale. (DOCX) [file pone.0058663.s001.docx]

Table S1: United States Acculturation Scale

United States Acculturation Scale: A Measure to Assess Americanization

Jacobo Kupersztoch, Heather Stieglitz, Elizabeth Paulk, Holly G. Prigerson

Please specify non-English language: ______________________________ N/A

Please specify non-American country of origin: ________________________ N/A

|  | Non-English only | Mostly non-English, some English | English and non-English equally [bicultural] | Mostly English, some non-English | English only |
| --- | --- | --- | --- | --- | --- |
| 1. What language do you speak most often? | 1 | 2 | 3 | 4 | 5 |
| 2. What language do you prefer speaking? | 1 | 2 | 3 | 4 | 5 |
| 3. What language do most of your friends speak? | 1 | 2 | 3 | 4 | 5 |
| 4. The music you listen to most is in | 1 | 2 | 3 | 4 | 5 |
| 5. The music you enjoy most is in | 1 | 2 | 3 | 4 | 5 |
| 6. The TV shows you watch most are in | 1 | 2 | 3 | 4 | 5 |
| 7. The TV shows you prefer watching most are in | 1 | 2 | 3 | 4 | 5 |
| 8. The movies you watch most are in | 1 | 2 | 3 | 4 | 5 |
| 9. You are most comfortable reading (newspapers, books, magazines) in | 1 | 2 | 3 | 4 | 5 |
| 10. You are most comfortable writing in | 1 | 2 | 3 | 4 | 5 |
| 11. You think most often in | 1 | 2 | 3 | 4 | 5 |
|  | Non-American only | Mostly non-American, some American | American and non-American equally (bicultural) | Mostly American, some non-American | American only |
| 12. Your father’s cultural identity was or is  (country of origin) | 1 | 2 | 3 | 4 | 5 |
| 13. Your mother’s cultural identity was or is  (country of origin) | 1 | 2 | 3 | 4 | 5 |

|  | Non-American only | Mostly non-American, some American | American and non-American equally (bicultural) | Mostly American, some non-American | American only |
| --- | --- | --- | --- | --- | --- |
| 14. Your friends while you were growing up were of _______ origin | 1 | 2 | 3 | 4 | 5 |
| 15. Your family cooks/eats foods that are of _______ origin | 1 | 2 | 3 | 4 | 5 |
| 16. Your friends now are of _______ origin | 1 | 2 | 3 | 4 | 5 |
|  | Non-American only | Mostly non-American, some American | American and non-American equally (bicultural) | Mostly American, some non-American | American only |
| 17. You like to identify yourself as | 1 | 2 | 3 | 4 | 5 |
|  | <2 times/yr | >4 times/yr | Monthly | Weekly | Daily |
| 18. Your contact (letters, phone calls, emails) with (country of origin) has been | 1 | 2 | 3 | 4 | 5 |
| 19. Where would you want to be buried? | 1. (Non-United States) country of origin | |  | 5. U.S. | |

20. Rater Assessment: How acculturated to the US (Americanized) do you think this respondent is?

Completely Americanized - 5

Mostly Americanized - 4

Bicultural - 3

Mostly non-American - 2

Completely non-American - 1
